# Effect of pH and lipopolysaccharide on tight junction regulators and inflammatory markers in intestinal cells as an experimental model of weaning transition in dairy calves

**DOI:** 10.3168/jdsc.2022-0333

**Published:** 2023-07-21

**Authors:** B.C. Agustinho, A.E. Mark, A.H. Laarman, D.E. Konetchy, P. Rezamand

**Affiliations:** a Department of Animal, Veterinary and Food Sciences, University of Idaho, Moscow 83844; b Agricultural, Life and Environmental Sciences, University of Alberta, Edmonton, AB, Canada T6G2P5

## Abstract

Acidic conditions combined with the presence of lipopolysaccharide (LPS) may increase the permeability of gastrointestinal epithelium. Feeding starch-rich starter to dairy calves is associated with ruminal acidosis and decreases the pH of other segments of the gastrointestinal tract, and that affects the tight junction regulator. The objective was to evaluate the effect of the combination of different pH (7.4 vs. 6.0) and LPS concentrations (0, 0.5, 10 ng/mL) in intestinal cells on tight junction regulators, inflammatory markers, and permeability. The human colon carcinoma Caco-2 cell line was used with the main treatment of pH and LPS in a 2 × 3 factorial arrangement. The pH was acidic (pH 6.0) or physiologic (pH 7.4), whereas LPS was 0, 0.5, or 10 ng/mL. After cells reached 70%–80% of confluence, the media were replaced with each respective treatment medium. Cells were treated for 3 h for mRNA abundance analysis, 3 and 6 h for protein abundance determination, and 3, 6, 12, and 24 h for permeability determination. Protein abundance of the myosin light-chain kinase (MYLK) and toll-like receptor 4 (TLR4) were measured by western blot. The mRNA abundance of IL-8, MYLK, peroxisome proliferator activated receptor gamma, and nuclear factor kappa B (Nfkb1) was determined by real-time, quantitative PCR. Paracellular permeability was determined with Lucifer yellow after 21 d of incubation. Cell culture was performed in biological triplicate; each biological replicate for real-time, quantitative PCR had 2 technical replicates, and for protein abundance and permeability assay had one technical replicate. The MIXED procedure of SAS (SAS Institute Inc.) was used with LPS, pH, and pH × LPS as fixed effects. Significance was declared at *P* ≤ 0.05 and tendencies when 0.05 < *P* ≤ 0.10. Increasing LPS doses did not affect the protein abundance of MYLK and TLR4, nor mRNA abundance of MYLK and PPRG. The LPS tended to increase mRNA abundance of IL-8 while pH × LPS interactively increased mRNA abundance of Nfkb1, where mRNA abundance of Nfkb1 was lower in cells exposed to pH 6.0 when combined with 0 and 10 ng/mL of LPS. Contrary to expectations, LPS did not affect the permeability of Caco-2 cells. The mRNA abundance of MYLK was greater at pH 6.0 versus pH 7.4. Also, protein abundance of TLR4 was lower at pH 6.0 than pH 7.4, and it decreased when exposure increased to 6 h. In addition, mRNA abundance of IL-8 was lower at pH 6.0 versus pH 7.4. Permeability was greater at pH 6.0 versus 7.4 after 6, 12, and 24 h of treatment. In summary, the effect of LPS and its interaction with pH showed less impact than expected on dependent variables measured, which might be attributed to the adopted clinically achievable LPS doses likely not being high enough to draw a strong response as observed in the literature. On the other hand, pH was far more relevant, modulating mRNA abundance of inflammatory markers, tight junction regulators, and permeability in in vitro colon cell models.

## Highlights

• Extended period of acidic pH can increase LPS concentrations and lead to local and systemic inflammation in the cattle gastrointestinal tract.
• The present study showed that LPS increased inflammatory marker (IL-8) mRNA abundance.
• Acidic pH decreased TLR4 and MYLK protein abundance.
• Acidic pH also increased tight junction permeability in Caco-2 colon cells after 12 and 24 hours of exposure.

## Graphical Abstract

**Figure:**
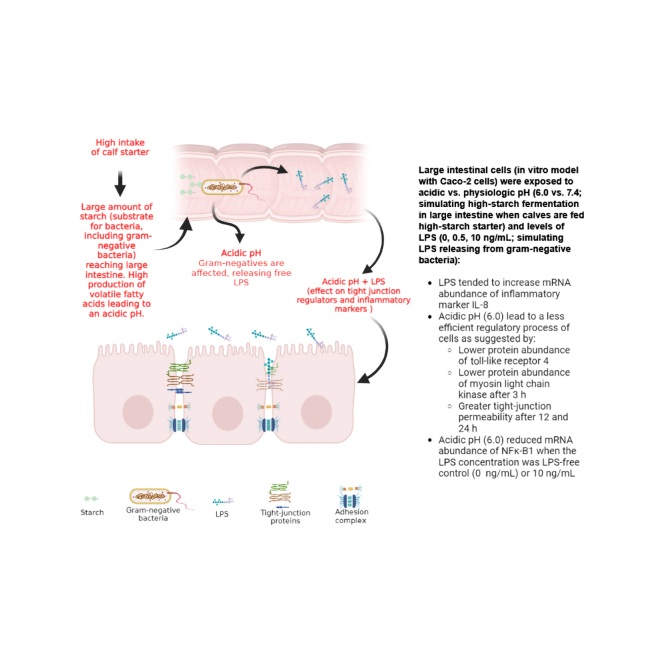
**Summary:** The weaning process in dairy calves is characterized by a rapid increase of readily fermentable carbohydrates, such as starch, which can lead to high production of volatile fatty acids. This could consequently lead to reducing the rumen pH, as well as increasing the amount of starch reaching the large intestine. A greater amount of starch in the large intestine possibly enhances local fermentation and lipopolysaccharide (LPS) concentration. Lipopolysaccharide has shown the potential to impact gastrointestinal epithelial permeability that leads to an inflammatory response, especially when the pH is low. The current study showed that LPS and acidic pH in large intestinal cells (simulating the large intestine of dairy calves) tended to show increased mRNA abundance of inflammatory markers such as interleukin-8 (IL-8), and cause a less efficient regulatory process of cells, respectively. However, acidic pH with no LPS or addition of 10 ng/mL of LPS reduced the mRNA abundance of NFκ-B1.

In dairy calf rearing, weaning is characterized by a rapid increase of readily fermentable carbohydrates (NASEM, 2021), leading to high production of VFA and low rumen pH. This high production of VFA is important for rumen papillae development (Drackley, 2008) and needed for postweaning nutrient absorption, though the concurrent low rumen pH may also physiologically affect the gastrointestinal tract (**GIT**). In adult cows, extended periods of depressed rumen pH, below 5.8, is characterized as SARA, a condition associated with increased LPS concentration in the rumen. Surges in LPS concentrations not only damage the rumen epithelial tight junctions but increase permeability in the rumen and colon (Emmanuel et al., 2007), leading to a local and potential systemic inflammation (Khafipour et al., 2009), with numerous adverse health effects (Plaizier et al., 2008).

Despite the well-characterized response of adult cows to LPS surges and low pH, the luminal conditions in calf GIT remain poorly understood. Although calves experience depressed rumen pH extensively before and during the weaning transition, they can maintain growth and health during this period (Laarman and Oba, 2011; Laarman et al., 2012; Kim et al., 2016). In yearling steers, low rumen pH is linked to depressed intestinal pH (Pederzolli et al., 2018), attributed to the an increased fermentation in the colon. Low intestinal pH in humans has been associated with the incidence of inflammation, such as bowel disease (Nugent et al., 2001; Tcymbarevich et al., 2019). Also, Eckert et al. (2015) have shown that calves fed more than 1.5 kg of started per day increased the fecal starch, which indicates a greater concentration of starch in the colon (Fischer et al., 2019), possibly increasing local fermentation and, depending on the severity, leading to colon acidosis and increased concentration of LPS. Furthermore, LPS has shown the potential to increase gastrointestinal epithelial permeability, especially in the colon, when the pH is acidic (Emmanuel et al., 2007). The cellular mechanisms and key pathways involved in the inflammatory and permeability response to low pH and high LPS are unclear and may hold important information on how to promote gastrointestinal health during weaning, especially in the colon, where there is an high knowledge gap. Therefore, the objective of the present study was to evaluate the effect of the combination of different LPS concentrations (0, 0.5, 10 ng/mL of LPS) in physiologic and acidic pH (pH 7.4 and 6.0) in large intestinal cells on tight junction regulators, inflammatory markers, and permeability in cell culture, as an experimental model to intestinal response in the early life of dairy calves.

The human colon carcinoma Caco-2 cell line (ATCC, Manassas, VA) was maintained in a culture medium composed of Dulbecco's Modified Eagle Medium with 4.5 mg/mL glucose, 50 U/mL penicillin, 50 U/mL streptomycin, 4 mmol/L glutamine, 25 mmol/L HEPES, and 10% fetal bovine serum at 37°C, as described by Nighot et al. (2017). The experiment was carried out on cells between passages 30 to 35.

The experimental design consisted of 2 main factors pH and LPS in a 2 × 3 factorial arrangement of treatments, totaling 6 treatments, in a complete randomized design. The pH treatments were acidic (**A**; pH 6) or physiological (**P**; pH 7.4), whereas the LPS treatments were 0, 0.5, and 10 ng/mL, corresponding to control, low, and high LPS concentrations (**CL**, **LL**, and **HL**, respectively). Therefore, the treatments were pH 6.0 and 0 ng LPS/mL (**A-CL**), pH 6.0 and 0.5 ng LPS/mL (**A-LL**), pH 6.0 and 10 ng LPS/mL (**A-HL**), pH 7.4 and 0 ng LPS/mL (**P-CL**), pH 7.4 and 0.5 ng LPS/mL (**P-LL**), and pH 7.4 and 10 ng LPS/mL (**P-HL**). The LPS from *E. coli* O111:B4 (Sigma-Aldrich) was used as the LPS source. The LPS was reconstituted in PBS and adjusted according to the desired concentration.

Cell culture was performed in biological triplicate; mRNA abundance had 2 technical replicates per biological replicate, while protein and permeability analyses had a single technical replicate. Cells were seeded in 6-well plates at 7.5 × 10^5^ cells in each well and were allowed to grow until reaching a confluence of approximately 70% to 80% before treatment, corresponding to approximately 24 h. For permeability experiments, cells were seeded at a density of 1 × 10^5^ cells/cm^2^ onto permeable membrane supports (PET, 0.4 µm pore size; Falcon Brands) in 12-well plates and allowed to grow on well inserts for 21 d to allow for polarization. Cell medium was refreshed every 48 h during the 21 d of incubation.

After cells reached confluence, the medium was carefully replaced with the respective medium for each treatment. Cells were exposed to the treatment for 3 h for analysis of mRNA abundance, 3 and 6 h for protein abundance analysis, and 3, 6, 12, and 24 h for permeability determination. Following each experimental treatment, cells were washed with PBS (Gibco – Thermo Fisher Scientific Inc.). For Western blot experiments, cells were collected by scraping with 1 mL of ice-cold PBS. Cells were lysed using RIPA buffer; total protein concentration in each lysate was normalized using the Bradford assay (Bio-Rad Laboratories), and all the samples were adjusted to 2 mg/mL total protein. Proteins were separated using SDS-PAGE, then transferred to a PVDF membrane (0.2 µm pore; Invitrogen-Thermo Fisher Scientific Inc.) using a wet-transfer system. Membranes were blocked in 5% skim milk powder in 1× Tris-buffered saline with Tween 20 (**TBST**) for 1 h then incubated in primary antibody overnight at 4°C [myosin light-chain kinase (MYLK), 1:1,000 in blocking buffer, M7905, Sigma-Aldrich; toll-like receptor 4 (TLR4), 1:500 in blocking buffer, SC-293072, Santa Cruz Biotechnology; and β-actin, 1:500 in blocking buffer, SC-477778, Santa Cruz Biotechnology]. Membranes were washed with TBST, incubated in secondary antibody (Mouse HRP conjugate, Santa Cruz Biotechnology; 1:1,000 in TBST for MYLK and TLR4, and 1:2,000 in TBST for β-actin) for 1 h at room temperature, and washed again before detection. Chemiluminescence was measured using a Bio-Rad ChemiDoc (Bio-Rad Laboratories) with Pierce ECL Western Blotting Substrate (Pierce-Thermo Fisher Scientific Inc.). The protein band was analyzed using the software ImageJ (National Center for Biotechnology Information). All the data had the pixel density inverted, corrected to the background, and were expressed in relative ratio to the housekeeping protein.

For real-time quantitative PCR (**RT-qPCR**) experiments, cells were collected by trypsinization, and pellets washed to remove any excess trypsin. The RNA was extracted using Qiagen RNeasy Kits (Qiagen Science), and RNA quantity was measured with a spectrophotometer (ND-1000, Nanodrop Technologies). Synthesis of cDNA was performed by reverse transcription, using 1.5 μg of RNA template in a total volume 40-μL reaction. DNase I (Ambion Inc., Life Technologies) was used to remove any potential DNA contamination, and RT was done using Superscript II (Invitrogen-Thermo Fisher Scientific Inc.). The RT-qPCR was performed using PowerSYBR Green Master Mix (Invitrogen-Thermo Fisher Scientific Inc.), and the primer set for IL-8 (forward primer GGGTTGTGGAGAAGTTTTTG, reverse primer CAGACCCACACAATACATGA, melting temperature of 61.4 and 60.0°C, respectively; amplicon size of 136 nt), MYLK (forward primer GGGGACTTTCAGCCTTGT, reverse primer CTGCTTCGCAAAACTTCCT, melting temperature of 62.2 and 62.5°C, respectively; amplicon size of 133 nt), peroxisome proliferator activated receptor gamma (PPARG; forward primer TTCTGCATTCTGCTTAATTCC, reverse primer TTTCGTTAAAGGCTGACTCT; melting temperature of 61.2 and 58.7°C, respectively; amplicon size of 108 nt), and nuclear factor kappa B (Nfkb1; forward primer TACTCTGGCGCAGAAATTAG, reverse primer CTTCAATTGCTTCGGTGTAG, melting temperature of 60.1 and 60.4°C, respectively; amplicon size of 161 nt). Ribosomal protein lateral stalk subunit P1 (RPLP; forward primer CGTCCTCGTGGAAGTGAC, reverse primer TAGTTGGACTTCCAGGTCG, melting temperature of 62.6 and 60.8°C, respectively; amplicon size of 109 nt) and peptidylprolyl isomerase A (PPIA; forward primer CCGAGGAAAACCGTGTAC, reverse primer GTCTGCAAACAGCTCAAAG, melting temperature of 61.1 and 59.3°C, respectively; amplicon size of 133 nt) were used as housekeeping genes. Cycle threshold (**Ct**) of target genes was corrected by Ct of housekeeping genes, delta Ct (ΔCt).

To evaluate the intestinal barrier function, the paracellular permeability of Lucifer yellow (LY) was analyzed according to procedure described by the manufacturer (Sigma-Aldrich) in the technical bulletin (catalog number: MTOX1002PC24) with modifications. The cells were exposed to the respective treatments for 3, 6, 12, and 24 h. Samples were removed, and the remaining liquid from apical and basal wells was aspirated. A portion of the aspirated was used for analysis, and the rest was discarded. The percentage of permeability was calculated according to the manufacturer, following this equation:

% permeability = 100 × (sample − blank)/(Lucifer yellow − blank).

All statistical analyses were carried out using the MIXED procedure of SAS (version 9.4, SAS Institute Inc.). The model consisted of pH, LPS concentration, and pH × LPS interaction as the fixed effects and well position in the plate as a random effect for the data regarding mRNA abundance. Protein abundance and permeability were analyzed as repeated measurements, and the model consisted of pH, LPS concentration, time, pH × LPS interaction, pH × time interaction. The mRNA abundance data were analyzed using ΔCt values and converted to fold change using the 2−^ΔΔCt^ method to present the results as graphs. Covariance structures were tested for repeated measures and chosen based on the lowest Akaike's information criterion. Interactions were investigated using the slice option. Differences were declared significant at *P* ≤ 0.05 and tendencies when 0.05 < *P* ≤ 0.10. Data presented are least squares means ± standard error of the means unless indicated otherwise.

The actin cytoskeleton is responsible for the epithelial shape of the cells and constitutes the peri-junctional actomyosin ring in the intestinal tissue, being physically related to the tight junction (Turner, 2000). Additionally, MYLK is well known for phosphorylating the actomyosin ring and causing the contraction, affecting the tight junction permeability (Shen et al., 2006; Weber et al., 2010). Therefore, the present study evaluated the effect of acidic and physiologic pH and different LPS concentrations on mRNA abundance of MYLK and protein abundance as a marker for tight junction regulators. However, the increasing LPS concentrations used did not affect the protein abundance of MYLK (*P* = 0.44, Figure 1, panel A) or the mRNA abundance of MYLK (*P* = 0.72, Table 1).

**Figure 1:**
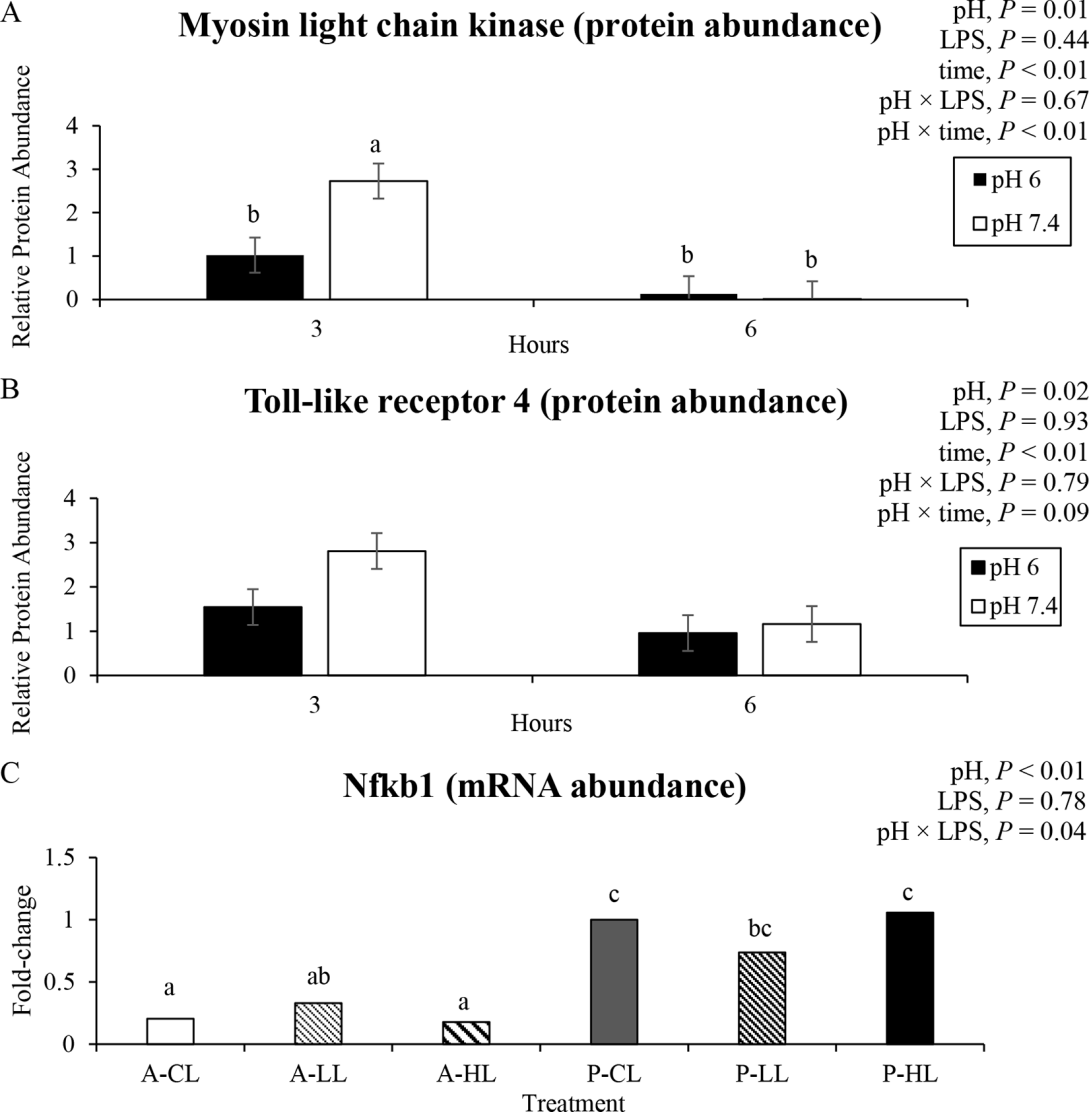
Relative protein abundance of myosin light-chain kinase (panel A), toll-like receptor 4 (panel B) in Caco-2 cells exposed to different pH and LPS concentrations after 3 and 6 h, and relative mRNA abundance expressed in fold change of nuclear factor kappa B (Nfkb1) gene (panel C). A-CL = pH 6.0 and 0 ng LPS/mL; A-LL = pH 6.0 and 0.5 ng LPS/mL; A-HL = pH 6.0 and 10 ng LPS/mL; P-CL = pH 7.4 and 0 ng LPS/mL; P-LL = pH 7.4 and 0.5 ng LPS/mL; P-HL = pH 7.4 and 10 ng LPS/mL. Means with different letters (a–c) differ by Tukey *t*-test (*P* ≤ 0.05). mRNA abundance is presented in fold changes, but statistical analyses were conducted using delta cycle threshold (Ct). Error bars represent SEM.

**Table 1:** Relative mRNA abundance expressed in delta cycle threshold values (Ct target gene − Ct housekeeping gene) of inflammatory markers and myosin light-chain kinase in Caco-2 cells exposed to different pH and LPS concentrations

Item^1^	pH^2^		LPS^3^			SEM	*P*-value		
	A	P	CL	LL	HL		pH	LPS	pH × LPS
MYLK	8.19	9.73	8.89	9.21	8.77	0.392	<0.01	0.72	0.60
IL-8	9.01	6.20	8.13	7.71	6.98	0.322	<0.01	0.07	0.28
PPARG	8.29	7.73	7.97	8.02	8.04	0.279	0.11	0.99	0.66

1 MYLK = myosin light-chain kinase; IL-8 = interleukin-8; PPARG = peroxisome proliferator activated receptor gamma.

2 A = acidic pH (pH 6.0); P = physiological pH (pH 7.4).

3 CL = control LPS (0 ng/mL); LL = low LPS (0.5 ng/mL); HL = high LPS (10 ng/mL).

Even though the cytokines have been associated with inducing the disruption of tight junction barriers by the modulation of MYLK (Al-Sadi et al., 2008; He et al., 2020), the increased mRNA abundance of IL-8 with increasing LPS concentration (*P* = 0.07) was likely, not severe at the point of modulating MYLK. The increased mRNA abundance of IL-8 was in agreement with Liu et al. (2016), who also observed increased mRNA abundance of IL-8 in Caco-2 cells when cells were treated with 100 ng/mL of LPS.

Proinflammatory response to LPS varied according to the variable as the following describes. Lipopolysaccharide is known to interact with receptors such as TLR4, thereby upregulating mRNA abundance of MYLK (Nighot et al., 2017) and activating proinflammatory pathways such as Nfkb1, triggering other expression of cytokines (Nyati et al., 2017). The LPS-free control and treatment with the LPS at 10 ng/mL reduced mRNA abundance of Nfkb1 at pH 6.0 only (*P* = 0.04, Figure 1, panel C). While the response at 10 ng/mL was likely related to a proinflammatory response, the reduced mRNA abundance of Nfkb1 at 0 ng/mL LPS was unexpected. Further investigation is required to better understand why the same was not observed for the 0.5 ng/mL of LPS. Furthermore, increasing doses of LPS did not affect the protein abundance of TLR4 (*P* = 0.93) or the mRNA abundance of PPRG (*P* > 0.72), a nuclear receptor that play the whole as transcription factor associated with synthesis of anti-inflammatory cytokines (Martin, 2010).

Despite the increased mRNA abundance of IL-8 gene in response to LPS concentrations, LPS did not affect the permeability of Caco-2 cells (*P* = 0.59, Figure 2). We believe this occurred because even though IL-8 tended to have a greater mRNA abundance with LPS, this probably was not enough to affect the tight junction regulators, such as MYLK, and potentially result in a greater permeability. Concentrations of LPS used to induce increased permeability have been much higher, corresponding to 500 µg of LPS/mL (50,000 times the highest concentration in this present study; Emmanuel et al., 2007), so the threshold at which LPS begins to have more consistent and sustained physiological responses still requires further investigation.

**Figure 2:**
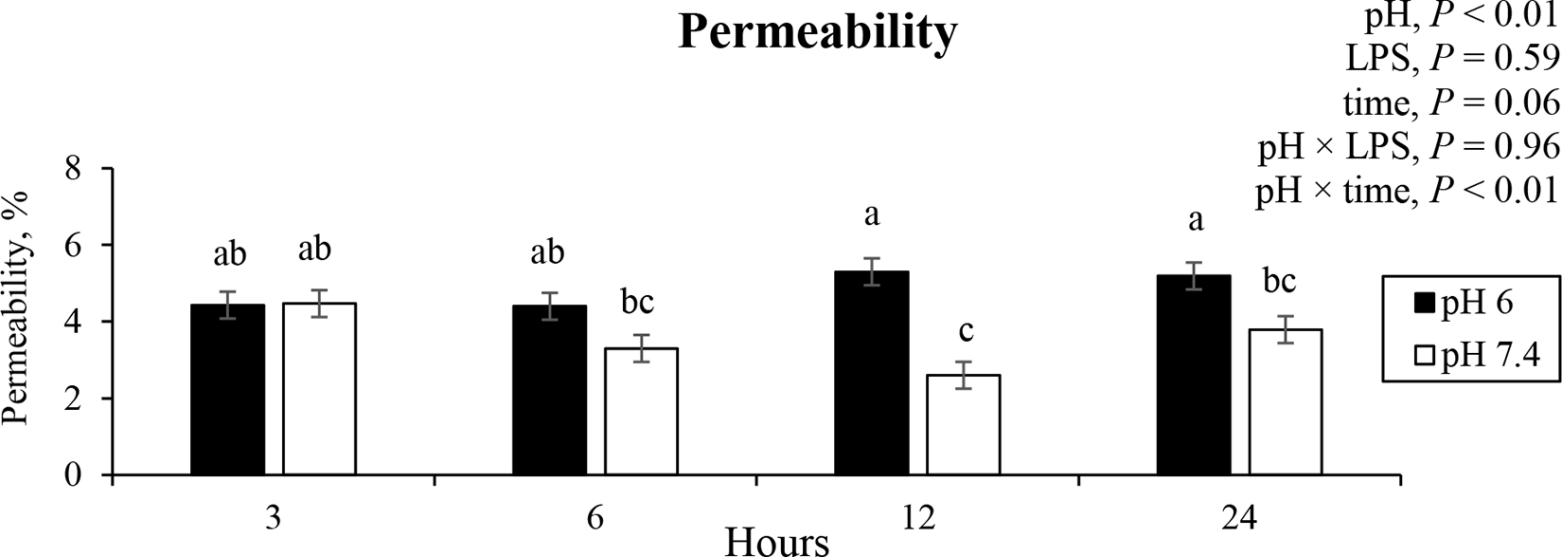
Permeability of Caco-2 cells exposed to different pH (acidic or physiologic) and LPS concentrations (0, 0.5, or 10 ng/mL) after 3, 6, 12, and 24 h. Means with different letters (a–c) differ by Tukey *t*-test (*P* ≤ 0.05). Error bars represent SEM.

On the other hand, pH played an important role in regulating inflammatory markers and tight junction regulators in our study. Abundance of TLR4 protein was lower at pH 6.0 than at pH 7.4 (*P* = 0.02, Figure 1, panel B) and generally decreased when the time of exposure increased from 3 to 6 h (*P* < 0.01). In addition, mRNA abundance of IL-8 was lower in cells exposed to pH 6.0 than pH 7.4 (*P* < 0.01, Table 1). Further, cells exposed to pH 6.0 for 3 h or pH 6.0 and 7.4 for 6 h had a lower abundance of MYLK protein than pH 7.4 for 3 h (*P* < 0.01, Figure 1). In contrast, mRNA abundance of MYLK was greater in cells exposed to luminal pH 6.0 than pH 7.4 (*P* < 0.01). The reduction in the abundance of TLR4, mRNA abundance of IL-8, and abundance of MYLK in pH 6.0 after 3 h of exposure suggests less efficient cell functional regulation in acidic pH (6.0). Although it is unknown which pathway induced the reduction of TLR4 protein in pH 6.0 compared with pH 7.4, Nyati et al. (2017) reported that TLR4 is responsible for triggering the expression of inflammatory cytokines, possibly explaining the lower mRNA abundance of IL-8.

Even though the literature reported that high LPS concentration combined with an acidic pH environment in the GIT increased permeability (Emmanuel et al., 2007), there was no effect of pH × LPS interaction or effect of LPS concentration on permeability detected under our experimental conditions (*P* = 0.96 and 0.59, respectively). This difference might be attributed to the higher LPS doses (50,000 times the dose used in the present study) and lower acidic pH (5.5) adopted by Emmanuel et al. (2007).

There was an effect of pH × time interaction on the permeability (*P* < 0.01), where permeability was reduced when the cells were exposed to pH 6.0 versus pH 7.4 for 12 and 24 h. We speculate that this observation is likely attributed to another factor not evaluated in the present study because this observation followed an opposite pattern compared with the other variables related to the integrity of the epithelial barrier. Several regulators modulate the permeability, and this study might not have evaluated the regulator that led to the increased permeability (Liu et al., 2012). In agreement with our observation, Pederzolli et al. (2018) reported varied responses to ruminal acidosis on gene expression of tight junction regulators in the jejunum in steers, where some tight junction regulators had an increased expression, and others did not change.

In summary, the effect of LPS and pH interaction and LPS alone showed less impact on the tight junction regulators, inflammatory markers, and permeability than expected, which might be attributed to the adopted clinically achievable LPS doses, that were not as high and effective as observed in other studies from the literature. On the other hand, pH was far more relevant, modulating mRNA abundance of inflammatory markers, tight junction regulators, and permeability, possibly suggesting a less efficient cell functional regulation in acidic pH. Time of exposure also differently affected permeability and mRNA abundance of inflammatory markers, providing an avenue for investigating tolerance to low pH for immune responses in the colon of dairy calves.
